# CISD3 inhibition drives cystine-deprivation induced ferroptosis

**DOI:** 10.1038/s41419-021-04128-2

**Published:** 2021-09-08

**Authors:** Yanchun Li, Xin Wang, Zhihui Huang, Yi Zhou, Jun Xia, Wanye Hu, Xu Wang, Jing Du, Xiangmin Tong, Ying Wang

**Affiliations:** 1 Laboratory Medicine Center, Clinical Research Institute, Zhejiang Provincial People’s Hospital, Affiliated People’s Hospital, Hangzhou Medical College, Hangzhou, Zhejiang 310014 China; 2 Department of Central Laboratory, Affiliated Hangzhou first people’s Hospital, Zhejiang University School of Medicine, Hangzhou, Zhejiang 310006 China; 3 College of Pharmacy, Hangzhou Normal University, Hangzhou, Zhejiang 311121 China; 4 Laboratory Medicine Center, Department of Laboratory Medicine, Zhejiang Provincial People’s Hospital, Affiliated People’s Hospital, Hangzhou Medical College, Hangzhou, Zhejiang 310014 China; 5 Bengbu Medical College, Bengbu, Anhui, 233000 China; 6 Phase I Clinical Research Center, Zhejiang Provincial People’s Hospital, Affiliated People’s Hospital, Hangzhou Medical College, Hangzhou, Zhejiang 310014 China

**Keywords:** Oncogenes, Preclinical research

## Abstract

Ferroptosis, a new form of programmed cell death, not only promotes the pathological process of various human diseases, but also regulates cancer progression. Current perspectives on the underlying mechanisms remain largely unknown. Herein, we report a member of the NEET protein family, CISD3, exerts a regulatory role in cancer progression and ferroptosis both in vivo and in vitro. Pan-cancer analysis from TCGA reveals that expression of CISD3 is generally elevated in various human cancers which are consequently associated with a higher hazard ratio and poorer overall survival. Moreover, knockdown of CISD3 significantly accelerates lipid peroxidation and accentuates free iron accumulation triggered by Xc^–^ inhibition or cystine-deprivation, thus causing ferroptotic cell death. Conversely, ectopic expression of the shRNA-resistant form of CISD3 (CISD3res) efficiently ameliorates the ferroptotic cell death. Mechanistically, CISD3 depletion presents a metabolic reprogramming toward glutaminolysis, which is required for the fuel of mitochondrial oxidative phosphorylation. Both the inhibitors of glutaminolysis and the ETC process were capable of blocking the lipid peroxidation and ferroptotic cell death in the shCISD3 cells. Besides, genetic and pharmacological activation of mitophagy can rescue the CISD3 knockdown-induced ferroptosis by eliminating the damaged mitochondria. Noteworthily, GPX4 acts downstream of CISD3 mediated ferroptosis, which fails to reverse the homeostasis of mitochondria. Collectively, the present work provides novel insights into the regulatory role of CISD3 in ferroptotic cell death and presents a potential target for advanced antitumor activity through ferroptosis.

## Introduction

Ferroptosis is a newly described programmed cell death, typically characterized by free iron overload and lethal phospholipid peroxide generation [1, 2]. Currently, studies have screened erastin, RSL3, sorafenib, artemisinin, and other molecules for their ability to induce tumorigenic ferroptosis [3–5], and identified SLC7A11/xCT, glutathione peroxidase 4 (GPX4), nuclear factor erythroid 2-related factor 2 (NRF2), ACSL4 and LPCAT3 enzymes as the ferroptosis regulatory proteins [6]. Although the primary pathway of ferroptosis has been established, the specific molecular mechanism underlying this regulatory network remains largely unknown.

Mitochondria, the core organelle for energy metabolism, plays a pivotal role in the regulation of fatty acid, amino acid, iron, and carbon metabolism [7]. Plenty of evidence demonstrated that diverse cellular metabolic pathways in mitochondrion could trigger ferroptosis [8]. For instance, Minghui Gao et al. illustrated that the TCA cycle and mitochondrial electron transport chain involved in cystine-deprivation induced ferroptosis but not GPX4 inhibition induced ferroptosis [9]. Elsewhere, Daiha Shin et al. demonstrated that dihydrolipoamide dehydrogenase increased α-KG level via glutaminolysis and activated cystine-deprivation ferroptosis [10]. The iron−sulfur cluster is a highly ancient and conservative cofactor that is mainly assembled in mitochondrion [11]. A recent study demonstrated that cancer cells depend on high levels of the ISC biosynthesis, and suppression of NFS1 robustly triggered ferroptosis in conjunction with cystine/glutamate antiporter inhibitor [12]. Our previous work also revealed the dysfunction of Frataxin, the leading cause of Friedreich’s ataxia, acted as an essential regulator of ferroptosis through the induction of free iron overload and dysfunction of mitochondrial homeostasis [13]. Altogether, these observations strongly demonstrate that mitochondria play a central regulatory role in ferroptosis.

The highly conserved NEET family proteins are mainly located in mitochondria and play important role in human health and disease [14]. They are unique because the [2Fe−2S] cluster can be redox-activated by binding with the CDGSH motif [15, 16]. In humans, only three different genes are currently known to encode NEET proteins. The least studied is CISD3, also known as Miner2 or MiNT, which differs from the other two family members as it encodes a monomer containing two [2Fe−2S] CDGSH motifs. Current research suggests that CISD3 coordinates a complementary role in mitochondrial iron and ROS regulation within the mitochondrial matrix [17]. However, whether CISD3 plays a regulatory role in mitochondria homeostasis and ferroptosis remains elusive.

In this study, through a series of cellular, molecular, and pharmacological analyses, we demonstrate that the mitochondria-localized protein CISD3 exerts a crucial role in Xc^–^ inhibition or cystine-deprivation-induced ferroptosis. Mechanistically, we found that CISD3 depletion presents a metabolic reprogramming toward glutaminolysis, which is required for the generation of sufficient lipid ROS to initiate ferroptosis. Both the inhibitors of glutaminolysis and the ETC process were capable of blocking the lipid peroxidation and ferroptotic cell death in the shCISD3 cells induced by cystine-deprivation. Importantly, genetic and pharmacological activation of mitophagy could block the ferroptosis in shCISD3 cells by clearing the damaged mitochondria. Of note, we present the first study to uncover the role of CISD3 in molecular biological characteristics of cancer and present a potential target predisposing cancer cells to an increased risk of ferroptotic cell death.

## Materials and methods

### Reagents

Erastin, ferrostatin-1, Z-VAD-FMK, Necrosuifonamide, Rapamycin, Deferoxamine (DFO), BafA1 were obtained from Selleck Chemicals (Houston, TX). MitoQ, SKQ1, IKE, 3-MA were purchased from Medchem Express (MCE, USA). Rotenone, DBM, antimycin A, NaN3, CCCP, DCF-DA, N-acetylcysteine (NAC), and glutathione (GSH) were purchased from Sigma-Aldrich (Merck, Darmstadt, Germany). CCK-8 Assay Kit was obtained from Meilunbio (Dalian, China). The Cell Apoptosis detection kit was purchased from MultiSciences (Hangzhou, China). The BODIPY 581/591 C11, MitoTracker and MitoSOX probe were obtained from Invitrogen (Carlsbad, CA, USA).

### Cell culture

Human acute promyelocytic leukemia cell line HL60 was obtained from The Cell Bank of Chinese Academy of Sciences (Shanghai, China) and cultured in RPMI 1640 medium (Hyclone, Logan, UT, USA) containing 10% fetal bovine serum (Gibco, Grand Island, NY, USA), penicillin (100 U/mL) and streptomycin (100 μg/mL). Human fibrosarcoma HT-1080 cells and 293T cells were obtained from the Cell Bank of Chinese Academy of Sciences (Shanghai, China) and cultured in DMEM medium (Hyclone, Logan, UT, USA) containing 10% fetal bovine serum (Gibco, Grand Island, NY, USA), 100 U/mL penicillin and 100 μg/mL streptomycin. Cells were maintained in an incubator with a humidified atmosphere of 5% CO_2_ at 37 °C and were used within 20 passages.

### Edu incorporation assay

EdU (5-ethynyl-2′ -deoxyuridine) incorporation assay was used to test the cell proliferation, which was performed with the BeyoClick EdU Cell Proliferation Kit (Beyotime, Shanghai, China) in accordance with the manufacturer’s instruction. Representative images were viewed and captured under a confocal microscope.

### Iron assay

The mitochondrial chelatable iron pool was assessed using Rhodamine B-[(1, 10-phenanthroline-5-yl)-aminocarbonyl]benzyl ester (RPA), a Fe^2+^ specific fluorescent sensor. After treatment, cells were harvested and incubated with 2 μM RPA for 15 min at 37 °C in Hanks balanced salt solution (HBSS), then being washed subsequently three times with HBSS. Representative images were viewed and captured under a confocal microscope. Alternatively, fluorescent intensity was read at excitation/emission wavelength of 562/601 nm by microplate spectrophotometer and normalized to the corresponding cell number.

### Cell viability assays

Cells were seeded in 96-well plates (NEST Biotechnology) at a density of 2 × 10^4^ cells/well and cultured with erastin, IKE, or sorafenib for 12 h, cystine deprivation medium for 24 h, sulfasalazine for 48 h. After treatment, 10 μL Cell Counting Kit-8 (CCK-8) reagent was added to each well and incubated for 2 h at 37 °C. Absorbance was measured at 450 nm with a background correction at 650 nm using the microplate reader.

### PI staining

The cells were seeded into 96-well plates at a density of 2 × 10^4^ cells/well, and treated with the indicated concentrations of drugs for 12 h. Then, Propidium Iodide (PI, 10 µg/mL) was added to each well and incubated at 37 °C for 10 min in the dark. Images were obtained using a fluorescence microscope (Nikon, Japan).

### Fluorescent probes staining

An equal amount of cells were treated as designed and stained with the fluorescent probe of DCF-DA (5 μM), BODIPY (4 μM), and MitoSOX (3 μM), which were used for detecting the level of cellular ROS, lipid peroxides, and mitochondrial ROS, respectively. After incubation for 30 min at 37 °C, cells were washed three times with HBSS and subsequently detected by flow cytometry or confocal microscopy.

### Fractions isolation

Briefly, indicated cells were washed, trypsinized, and subjected to the subcellular fractions isolation kit (Beyotime, Shanghai, China). Mitochondrial, cytoplasm, and nucleus fractions were lysed and prepared for western blot analyses.

### Protein extraction and western blot analysis

After indicated treatment, cells were harvested and washed twice with ice-cold PBS and lysed in RIPA buffer (Beyotime, Shanghai, China) containing complete protease and phosphatase inhibitor (Thermo, Waltham, MA). The concentration of protein was quantified by the bicinchoninic acid protein assay kit (Thermo, Waltham, MA). Subsequently, equal amounts of protein were separated by SDS–PAGE and transferred to PVDF membranes. The membranes were blocked with 5% skim milk for 1 h and incubated with the primary antibodies at 4 °C overnight. After being washed with TBST three times, the membranes were incubated with an appropriate HRP-conjugated secondary antibody (Beyotime, Shanghai, China) for 1 h at 25 °C. The membranes were washed with TBST three times again, then the blots were visualized using a chemiluminescence detection kit ECL-PLUS. The primary antibodies used in this experiment were as follows: anti-CISD3 (developed in this study), anti- Histone H3 (CST, #3638, 1: 1000), anti-Tom20 (CST, #42406s, 1: 1000), anti-PINK1 (CST, #6946s, 1: 1000), anti-Parkin (CST, # 4211s, 1: 1000), anti-DRP1 (Proteintech, #12186-1-AP, 1: 1000), anti-MFN2 (Abcam, ab184247, 1: 1000), anti-β-actin (Abcam, ab8226, 1: 1000). IRP2 (Abcam, ab232994, 1: 1000), TFR (Santa Cruz, sc-32272, 1: 200), ACSL4 (Abcam, ab155282, 1: 1000), NDUFS1(Abcam, ab157221, 1: 2000), ATP5A (Abcam, ab176569, 1: 1000), SDHB (Abcam, ab175225, 1: 5000), UQCRFS1(Abcam, ab191078, 1: 5000), COX4 (CST, #11967, 1: 1000), β-actin was served as the loading control.

### Immunofluorescence analysis

Cells were cultured for 24 h on polylysine coated glass coverslips in 24-well plates (2 × 10^5^ cells/well) and followed by indicated treatment. Then, cells were fixed, permeabilized, blocked, and incubated with primary antibodies MDA (Abcam, ab6463, 1: 100), Tom20 (CST, #42406s, 1: 100), Lamp1 (CST, #9091s, 1: 100), CISD3 (1: 100) at 4 °C for overnight. Subsequently, cells were washed and incubated with the corresponding secondary antibodies at 37 °C for 1 h. The used secondary antibodies were Alexa Fluor 488 labeled anti-Rabbit IgG and Alexa Fluor 594 conjugated anti-mouse antibody (Abcam, Cambridge, MA). Cells were co-stained with 4′, 6-diamidino-2-phenylindole dihydrochloride (DAPI) (10 μg/mL) (Sigma, USA) for 10 min and visualized using laser confocal microscope (Leica, Germany).

### Transmission electron microscope (TEM)

After indicated treatments, the cells were fixed by 2.5% glutaraldehyde solution at 4 °C overnight. Subsequently, the samples were dehydrated by a graded series of ethanol and eventually transferred to absolute acetone. The following infiltration with absolute acetone and the final spurr resin mixture, the samples were embedded, ultrathin sectioned, and stained. Finally, the samples were observed in the Hitachi Model H-7650 TEM.

### Plasmids

For knockdown of *CISD3*, target shRNA sequences were subcloned into pLVX-shRNA Lentivector (Takara). The shRNA knockdown sequences for *CISD3* were as follows: 5′- GATCCGGCCTATCTCCACTCAAGTTCTTCAAGAGAGAACTTGAGTGGAGATAGGCCTTTTTTG-3′, and 5′- AATTCAAAAAAGGCCTATCTCCACTCAAGTTCTCTCTTGAAGAACTTGAGTGGAGATAGGCCG-3′. Full-length GPX4, FTH, Parkin, and CISD3 cDNA were ordered from Sino Biological (Beijing, China) and subcloned into pLVX-IRES-Neo lentivirus vector (Takara, Dalian, China) by ClonFast Seamless Cloning kit (obio, Nanjing, China). The shRNA-resistant form of CISD3 (CISD3res) was generated according to described methods by introducing silent changes in the coding region targeted by the shRNA [18]. All the recombinant lentiviral plasmids were verified by sequencing.

### Lentiviral packaging and transduction

The recombinant lentiviral plasmids were co-transfected with pMD2.G, pSPAX2 into 293T cells to produce recombinant lentiviral. Lentivirus infections were carried out as described previously [19]. Briefly, cells seeded in 24-well plates reached 70–80% confluence, and the complete DMEM medium was removed. Cells were then transfected with the corresponding lentivirus. After two days, G418 or puromycin was added for screening when the transfected cells reached 70% confluence. The stable cells were maintained in G418 or puromycin. The transfection efficiency was evaluated by western blot analysis.

### Fuel dependency assay

The Seahorse XF Mitochondrial Fuel Flex Test determines the rate of oxidation by measuring mitochondrial respiration in the presence or absence of fuel pathway inhibitors. The measurement of cells’ reliance on fuel pathways is tested by Agilent Seahorse XFe24 Analyzer in accordance with the manufacturer’s instructions. Briefly, sequentially inhibiting the pathways of glucose oxidation (UK5099, 2 μM), glutamine oxidation (C968, 10 μM), and long-chain fatty acid oxidation (Etomoxir, 4 μM) could calculate the dependence on glucose oxidation in meeting basic energy requirements. Glutamine oxidation dependency is tested by first injecting an inhibitor of glutamine oxidation, followed by inhibition of the other two alternative pathways.

### Glutamine oxidation stress test

The glutamine oxidation stress test was also analyzed by Agilent Seahorse XFe24 Analyzer. CISD3-silenced and control cells were seeded in a culture microplate and a sensor cartridge was hydrated in Agilent Seahorse XF Calibrant at 37 °C in a non-CO_2_ incubator overnight. The following day, cells were washed and incubated with warm Seahorse XF DMEM supplemented with 1 mM pyruvate, 2 mM glutamine, and 10 mM glucose, at 37 °C non-CO_2_ incubator for 1 h. Cells were subject to XF Substrates Oxidation Stress Tests and inject assay media (control) or C968, an allosteric inhibitor of glutaminase for the inhibition of glutamine oxidation pathway, followed by sequential injections of oligomycin (1.5 μM), FCCP (1.5 μM), and rotenone/antimycin A (0.5 μM). This method is ideally suited to the assessment for cellular substrate demand both under basal conditions, and in response to elevated substrate demand (maximal respiration).

### Tumor xenografts

4–6 weeks of nude mice were purchased from Shanghai SLAC Laboratory (Shanghai, China). 2.5 × 10^6^ HT-1080 cells with CISD3 knockdown or empty vector were suspended in 0.1 mL PBS and injected subcutaneously into the flank of nude mice. One week later, mice transplanted with control or CISD3-silenced cells were randomly allocated into two groups with the treatment of vehicle and erastin (30 mg/kg), respectively. The longest diameter (*a*) and shortest width (*b*) of tumors were measured every other day and tumor volume was calculated using the formula: 0.5 × *a* × *b*^2^. Two weeks later, the mice were sacrificed and the tumors were isolated and sliced for H&E and IHC staining. The animal experiment was approved by the Ethics Committee of Zhejiang Provincial People’s Hospital.

### Immunohistochemistry (IHC)

Tissues were fixed with 4% paraformaldehyde and embedded in paraffin. The paraffin-embedded block tissues were cut into 4 μm sections and followed dewaxed, hydrated, and antigen retrieval. After being washed with PBS, the slides were treated with 3% hydrogen peroxide for 15 min, then washed and blocked with 5% BSA for 15 min at room temperature. Subsequently, anti-CISD3 antibody (1:100), anti-Ki67 antibody (1:100), anti-4HNE antibody (1:100), and anti-MDA antibody (1:100) were added to the sections at 4 °C for overnight. The streptavidin peroxidase method was used for signal detection and then stained by diaminobenzidine (DAB) and counterstained with hematoxylin. The sections were observed and photographed under light microscope.

### Statistical analysis

The statistical significance of mean values was determined by an unpaired two-tailed Student’s *t*-test. The comparison of statistical significance among three or more groups was determined by one-way analysis of variance (ANOVA). Statistical analysis was performed using Graphpad Prism 7.0. *P* < 0.05 was considered to be significant.

## Results

### CISD3 expression is associated with cancer progression and iron metabolism

**Fig. 1:**
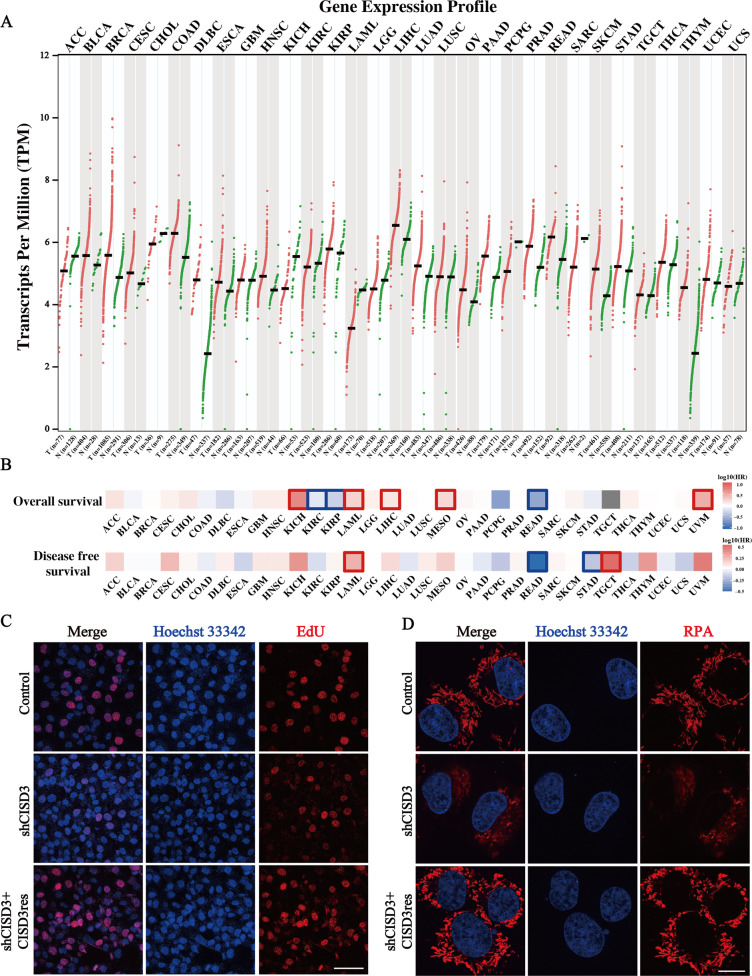
Association of CISD3 expression with cancer progression and iron metabolism. **A** Gene expression profile of CISD3 in various kinds of TCGA cancers and its homologous normal samples, which is obtained from the GEPIA website (http://gepia.cancer-pku.cn/index.html). **B** CISD3 expression was classified into two subgroups (high or low group) using the median level of CISD3 expression as the cut-off value. Survival map of Hazards Ratio (HR) for overall survival (OS) and disease-free survival (DFS) according to the low and high expression levels of CISD3 mRNA. **C** The proliferation of HT-1080 cells with different CISD3 expressions was detected by the EdU incorporation assay. Nuclei were visualized with Hoechst 33342. Scale bar: 50 μm. **D** Mitochondrial iron levels measured by RPA staining in control, shCISD3, and CISD3res HT-1080 cells and images were captured by confocal microscopy. Scale bar: 5 μm.

We wonder whether CISD3 could be associated with the molecular biological characteristics of cancer and be a potential indicator of tumor prognosis. First, the expression of CISD3 in multiple tumors and homologous normal samples was determined via the GEPIA website using samples from TCGA and GTEx projects (Fig. 1A). Higher expression of CISD3 was found in most cancers, such as BRCA, DLBC, LIHC, PRAD, SKCM, and THYM, and CISD3 expression exhibited a high hazard ratio in the overall survival (OS) and disease-free survival (DFS) of multiple cancer types (Fig. 1B). We performed a survival analysis of subgroups stratified by median values of CISD3 expression from the TCGA datasets. The results revealed that high CISD3 expression levels were correlated with poorer prognosis of OS in UVM (Log-rank *P* = 0.011, HR = 3.1), KICH (Log-rank *P* = 0.019, HR = 5.4), LAML (Log-rank *P* = 0.033, HR = 1.8), MESO (Log-rank *P* = 0.032, HR = 1.7), TGCT (Log-rank *P* = 0.041), and LIHC (Log-rank *P* = 0.036, HR = 1.4) (Fig. S1A). These results revealed that CISD3 is potentially associated with the progression of cancer in multiple tumor types.

To further verify the function of CISD3, stable CISD3 knockdown and shRNA-resistant CISD3 cDNA (CISD3res) overexpression HT-1080 cells were generated and verified by western blot (Fig. S1B). As expected, the silencing approach efficiently reduced the expression of CISD3 in the shRNA cells, whereas the shRNA-resistant CISD3 dramatically eliminated the knockdown effect and enhanced the protein level of CISD3. Using these cells, we found that CISD3 depletion did not cause cell death, but rather inhibited cellular proliferation (Fig. 1C). Conversely, overexpression of the shRNA-resistant form of CISD3 enhanced cellular proliferation as determined by EdU incorporation assay. Then, we used the selective yield fluorescence probe RPA to assess the cellular labile iron pool. The results showed that CISD3 suppression robustly activated the labile iron level with the decreased fluorescence of RPA, whereas enforced CISD3res expression blocked the accumulation of iron (Fig. 1D). This suggested that CISD3 is vital in maintaining iron homeostasis, an observation that is consistent with a previous study [17]. Thus, we proposed that CISD3 may play a regulatory role in cancer progression and iron metabolism.

### CISD3 is associated with cystine-deprivation induced ferroptosis

**Fig. 2:**
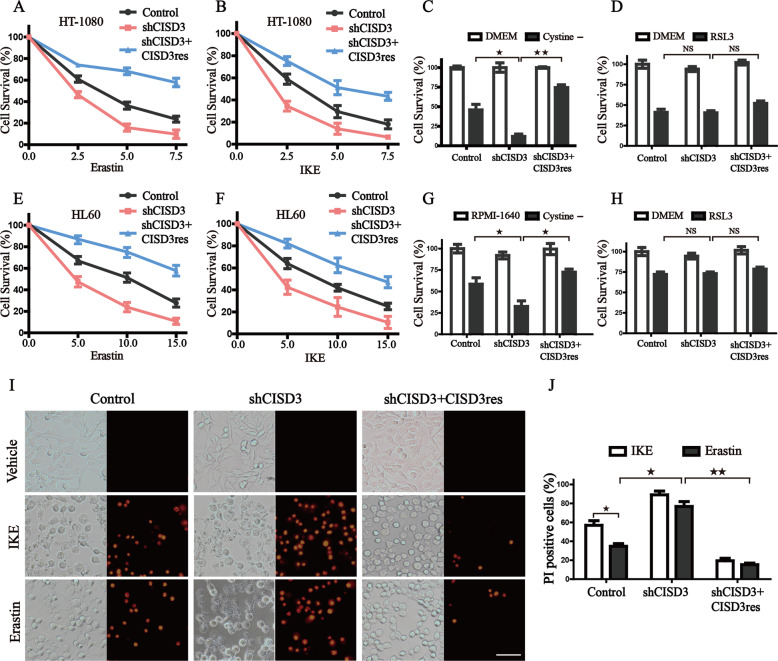
CISD3 linkage to cystine-deprivation induced ferroptosis. The HT1080 cells were exposed to titration of cystine/glutamate antiporter inhibitor erastin (**A**), and IKE (**B**) for 12 h, cystine-free media (**C**) for 24 h, or 0.5 μM RSL3 for 6 h (**D**), respectively. Cell survival was measured by CCK8 assay. Cell survival of HL-60 cells with different CISD3 expression was measured after exposure to indicated concentrations of erastin (**E**) and IKE (**F**) for 12 h, cystine-free media (**G**) for 24 h, or 0.1 μM RSL3 (**H**) for 12 h. **I, J** Indicated cells were exposed to 5 μM IKE or erastin for 12 h and subjected to PI staining. The images were captured by fluorescent microscopy and PI-positive cells were analyzed using Image J software. The scale bar in the cell image indicates 50 µm. The values are presented as means ± SD from three independent experiments. *P < *0.05*, **P < 0.01 versus control or between different groups with altered CISD3 expression.

Given the close association of free iron levels with ferroptosis, we hypothesized whether alteration of CISD3 expression might cause ferroptotic cell death. Therefore, HT-1080 cells with different expressions of CISD3 were treated with erastin, IKE, sulfasalazine, or cystine depletion medium, and cell survival was determined (Fig. 2A–C and S2). The results showed that CISD3 depletion significantly sensitized cells to cystine-deprivation-induced cell death. Conversely, transfection enforced expression of CISD3res could ameliorate the ferroptotic cell death. We observed a similar phenomenon in HL60 cells, indicating that CISD3 knockdown sensitized cells to cystine-deprivation ferroptosis, which was not restricted to a single cell lineage (Fig. 2E–G). But CISD3 expression failed to impinge on ferroptosis upon RSL3 treatment, demonstrating that GPX4 acts downstream of CISD3-mediates ferroptosis (Fig. 2D, H). Further, PI staining assay also verified that suppressing CISD3 expression accelerated the disruption of cell membrane permeability following erastin or IKE treatment (Fig. 2I, J). Collectively, these findings suggested that CISD3 knockdown facilitates cystine-deprivation-induced ferroptosis.

### CISD3 knockdown enhances the accumulation of free iron and lipid peroxides

**Fig. 3:**
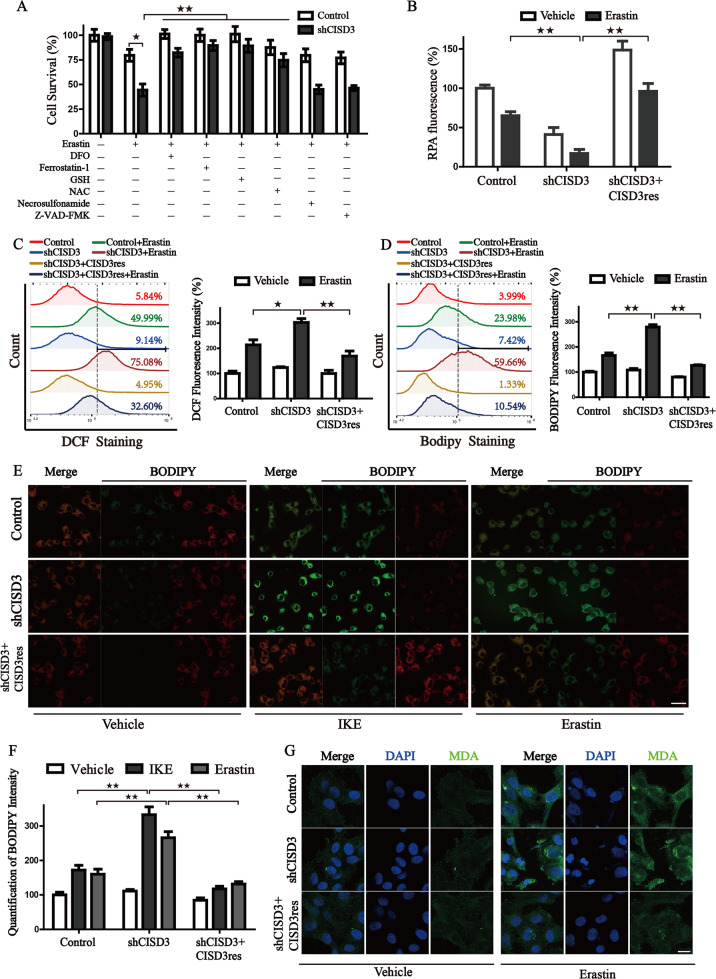
CISD3 Knockdown promotes the accumulation of lipid peroxides and free iron. **A** Control and CISD3 depletion cells were treated with DMSO or 7.5 μM erastin for 6 h in the presence or absence of several small molecular inhibitors (100 µM DFO, 1 µM ferrostatin-1, 0.5 mM GSH, 0.5 mM NAC, 0.5 µM Necrosulfonamide, 5 µM Z-VAD-FMK). The cell viability was detected by CCK8 assay. **B** Cellular ferrous ion levels were assayed by RPA staining in the cells exposed to 7.5 μM erastin for 6 h. The error bars represent the standard deviation from three replicates. HT1080 cells with different CISD3 expressions were treated with or without erastin (7.5 μM). Flow cytometric analysis of cellular ROS through DCF-DA staining (**C**) and cellular lipid peroxides through BODIPY C11 staining (**D**), corresponding statistical histograms are shown on the right. **E** Representative BODIPY C11 staining images of HT-1080 cells treated with or without Erastin (7.5 μM) and IKE (7.5 μM). A shift from red to green fluorescence indicates the accumulation of lipid peroxides. Scale bars: 30 μm. **F** Corresponding statistical histogram is shown. **G** HT-1080 cells were incubated with DMSO or erastin (7.5 μM) for 6 h, followed by immunofluorescence (IF) assay. The antibody against MDA was used to track the production of lipid peroxides. Nuclei were visualized with DAPI. Scale bar: 10 μm. *P < 0.05, **P < 0.01 versus control or between indicated groups.

To further characterize the mechanism of CISD3 knockdown mediated cell death, several inhibitors were used to examine the rescue effect on erastin induced cell death. Along with CISD3 silencing, the iron chelator (DFO), ferroptosis inhibitor (ferrostatin-1), and antioxidant (GSH, NAC) could rescue the cell death under erastin treatment. However, the inhibitor of necroptosis (Necrosulfonamide) and caspase inhibitor (Z-VAD-FMK) displayed no obvious impact on the viability recovery in the CISD3 depletion cells (Fig. 3A). But Fer-1 and NAC are incapable of boosting the growth rates of shCISD3 cells, demonstrating that antioxidant buffering itself may not be sufficient to generate the proliferation rescue (Fig. S3A). Then, cellular free iron level, one of the major hallmarks of ferroptosis, was monitored by RPA staining. Results indicated stable transduction of CISD3res blocked the accumulation of free iron in shCISD3 cells subjected to erastin (Fig. 3B). We next examined whether CISD3 mediated ferroptosis is associated with exacerbated peroxidation. Indeed, the increased fluorescence intensity of DCF was monitored in CISD3 deletion cells after erastin treatment, whereas enforced expression of CISD3res significantly blocked the accumulation of ROS (Fig. 3C). Besides, the formation of lipid peroxides was monitored by BODIPY staining and lipid metabolites detection. CISD3 knockdown increased the fluorescence of BODIPY, accelerated the staining of MDA, whereas CISD3res overexpression rescued the overload of lipid products under erastin exposure (Fig. 3D–G and S3B). These findings demonstrate that inhibition of CISD3 promotes ferroptotic cell death by accelerating lipid peroxidation and free iron accumulation.

### CISD3 plays a crucial role in the mitochondrial homeostasis

**Fig. 4:**
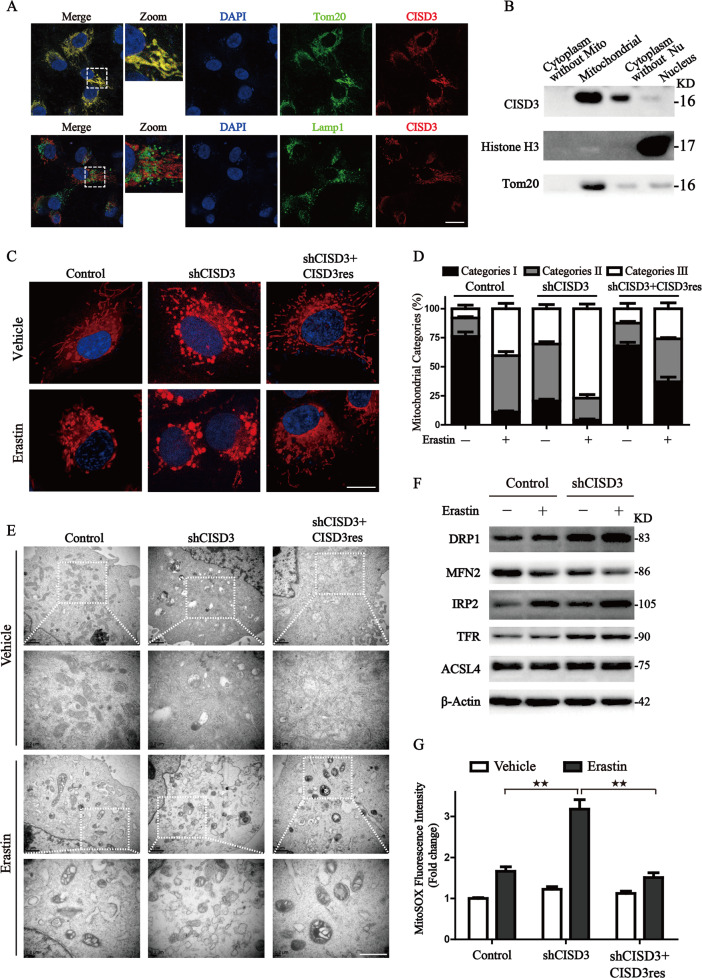
CISD3 plays a crucial role in the mitochondrial homeostasis. **A** Immunofluorescence assays verifying the localization of CISD3 protein in HT1080 cells. CISD3 protein was co-localized with mitochondrial protein Tom20, but not with lysosome marker, Lamp1. **B** Subcellular localization of the CISD3 protein was analyzed by western blot using protein extracted from purified mitochondrial, cytoplasm, and nucleus fractions. Antibodies against mitochondrial protein Tom20 and nucleoprotein Histone H3 were used as indicated markers. **C** Representative images of mitochondrial morphology labeled by MitoTracker Red in HT1080 cells with altered CISD3 expressions in the presence or absence of 7.5 μM erastin. Scale bars: 5 μm. **D** Mitochondrial morphology alteration was monitored through Mitotracker probe staining according to the degree of fragmentation. **E** The morphological changes of mitochondria were detected by transmission electron microscopy (TEM) in the absence or presence of 7.5 μM erastin. Lower scale bars: 1 μm. **F** HT1080 cells with scramble or shCISD3 vector were treated with or without 7.5 μM erastin for 8 h, the indicated protein levels were analyzed by western blot. β-actin served as the loading control. **G** Mitochondrial ROS production was detected by MitoSOX staining and measured by flow cytometry. The values are presented as means ± SD. *P < 0.05, **P < 0.01 versus control or between indicated groups.

The sequence of CISD3 contains a coding region of mitochondrial targeting transit peptide, predicting a mitochondrial localization. In this work, the specific subcellular localization of CISD3 was confirmed by the results from confocal microscopy and immunoblotting, which showed that CISD3 co-localized with mitochondrial protein Tom20, and expressed in the mitochondrial fraction (Fig. 4A, B). The abnormality of mitochondrial morphology has been demonstrated as another feature in ferroptotic cell death. We then labeled mitochondria with Mitotracker probe and found control cells showed a network of elongated mitochondria. On the contrary, mitochondria in shCISD3 cells appeared to be fragmented, indicating that CISD3 depletion destroyed mitochondrial homeostasis (Fig. 4C). To further address mitochondrial alterations, mitochondria were categorized into three classes according to the recently established classification system [20]. With the influence of erastin, the mitochondria in shCISD3 cells became disorganized, smaller, and networks collapsed around the perinuclear region (category III), whereas ectopic expression of CISD3res blocked the reduction of the proportion of category I−II (Fig. 4D). Furthermore, dramatic morphological changes of mitochondria were observed through transmission electron microscopy in CISD3 depletion cells, including mitochondrial fragmentation, vacuolization, and cristae enlargement, which were further aggravated under erastin treatment. Meanwhile, we observed that some of the damaged mitochondria formed double-membrane autophagosomes and eventually fused with lysosomes in the shCISD3 cells upon erastin treatment. Additionally, the results provided supportive evidence of the protective role of CISD3 in mitochondrial integrity maintenance under erastin treatment (Fig. 4E).

We next explored the process of mitochondrial dynamics for the abnormality of mitochondrial morphology. Results showed that mitochondrial fusion-associated protein Mfn2 was significantly reduced, whereas mitochondrial fission-associated protein DRP1 protein was increased in shCISD3 cells along with Xc^–^ inhibition. In addition, activation of IRP2 and TFR in the shCISD3 cells manifested the activation of iron-starvation stress and resulted in free iron overload (Fig. 4F). We further examined the mitochondrial ROS formation and the results illustrated that CISD3 knockdown stimulated the mitochondrial ROS accumulation, whereas CISD3res overexpression impeded the increased fluorescence of mitoSOX under the inhibition of system Xc^–^ (Fig. 4G). Collectively, these findings demonstrate the pivotal role of CISD3 in maintaining mitochondrial homeostasis and the balance of ROS metabolism.

### CISD3 depletion presents a metabolic reprogramming toward glutaminolysis

**Fig. 5:**
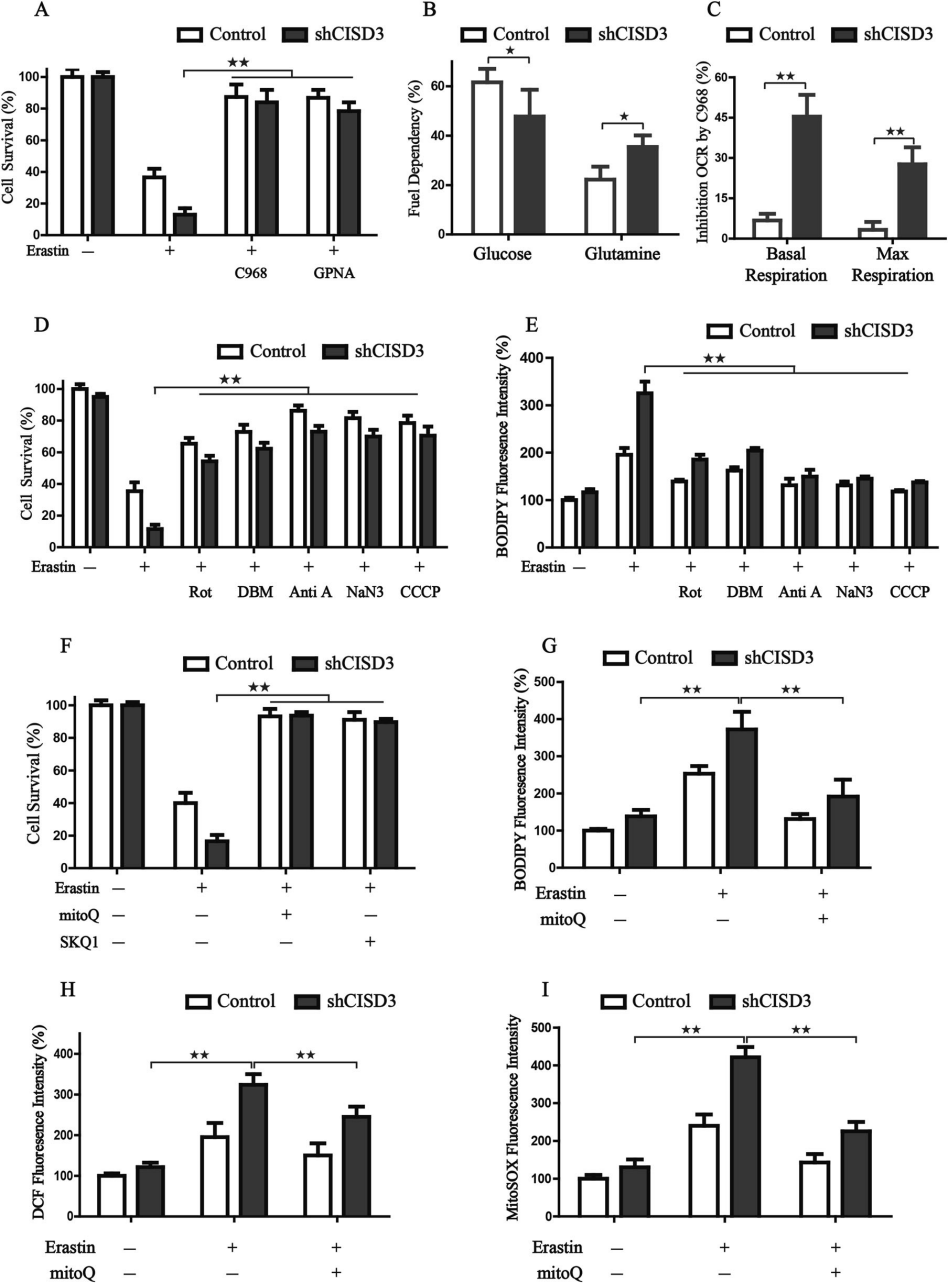
CISD3 depletion presents a metabolic reprogramming toward glutaminolysis. **A** Cell viability detection in control and CISD3-silenced cells at the presence or absence of indicated chemicals (7.5 µM Erastin, 10 µM C968, and 1 mM GPNA). **B** Fuel dependency assay for glucose and glutamine in control and CISD3-silenced cells. **C** After the glutamine oxidation pathway was inhibited by C968, the oxygen consumption (OCR) of basal and max respiration was monitored by Agilent Seahorse XFe24 Analyzer. The inhibition rate of OCR by C968 in control and CISD3-silenced cells was calculated. **D**, **E** Cell viability and cellular lipid peroxides detection in the control and CISD3-silenced cells at the presence or absence of indicated chemicals; (7.5 µM Erastin, 10 µM Rotenone (Rot), 2 mM DBM, 2.5 µM Antimycin A (Anti A), 15 mM NaN3, 5 µM CCCP). (F-I) Cell viability, lipid peroxides, cellular ROS, and mitochondrial ROS in control and CISD3-silenced cells were measured under the treatment of 7.5 µM erastin, at the presence or absence of 0.5 µM MitoQ or 0.2 µM SKQ1. The values are presented as means ± SD from three independent experiments. *P < 0.05, **P < 0.01 versus control or between different groups.

Previous studies demonstrated that mitochondria play a pivotal role in ferroptosis by fueling metabolic molecular, hyperpolarizing the MMP, and subsequently inducing lipid ROS production [9, 10]. Glutamine metabolism, known as glutaminolysis, is required for cysteine-deprivation-induced ferroptosis. We, therefore, examined whether glutaminolysis takes part in the ferroptosis mediated by CISD3 depletion. Indeed, when the glutaminolysis pathway was pharmacologically inhibited by C968 (GLS1 and GLS2 inhibitor) or GPNA (SLC1A5 inhibitor), the ferroptotic cell death was significantly prevented in CISD3 depletion cells (Fig. 5A). Further, we explore the influence of CISD3 on fuel dependency by Seahorse XFe24 Analyzer. Results showed that the silencing of CISD3 could reduce the dependency on glucose and increase the demand for glutamine oxidation pathway, indicating a conversion of energy supply in CISD3-silenced condition (Fig. 5B). In addition, when glutaminolysis was inhibited by C968, the OCR of basal respiration and max respiration in CISD3 depletion cells were significantly reduced, demonstrating that knockdown of CISD3 presents a metabolic reprogramming toward glutaminolysis (Fig. 5C). For the major function of glutaminolysis is to fuel the mitochondrial oxidative phosphorylation, various inhibitors of ETC and mitochondrial uncoupler CCCP was used to determine their efficiency in attenuating the ferroptosis process in CISD3 knockdown cells. Of note, inhibitors of mitochondrial complex I (rotenone), complex II (DBM), complex III (antimycin A), complex IV (NaN3), and MMP depolarization by CCCP restored cell viability and inhibited lipid peroxidation in shCISD3 cells cultured with Xc^–^ inhibitor (Fig. 5D, E). NaN3 and antimycin A exerted a more significant effect on eliminating lipid peroxidation for full inhibition of the ETC activity. Taken together, these findings indicate that ETC-associated oxidative metabolism fueled by glutaminolysis plays a crucial role in shCISD3 initiated cell death when cultured with Xc^–^ inhibitor.

Since CISD3 knockdown significantly triggered mitochondrial radicals generation induced by erastin, we determined whether the administration of mitochondria-targeted antioxidants would exert potential protective effects. Therefore, the lipophilic triphenylphosphonium-based ubiquinone analog mitoQ and its plastoquinone-containing analog SKQ1 were selected to test this hypothesis. Remarkably, both mitoQ and SKQ1 could almost completely neutralize the erastin induced ferroptotic cell death (Fig. 5F). Besides, mitoQ pretreatment could effectively inhibit the deleterious effects induced by erastin in shCISD3 cells, which were characterized by decreased lipid ROS accumulation and mitochondrial ROS production (Fig. 5G–I). Collectively, CISD3 depletion presents a metabolic reprogramming toward glutaminolysis and fuel the mitochondrial ETC, which results in oxidative stress and ferroptosis.

### MitoQ ameliorates CISD3 associated ferroptosis by regulating mitophagy and the process of mitochondrial dynamics

**Fig. 6:**
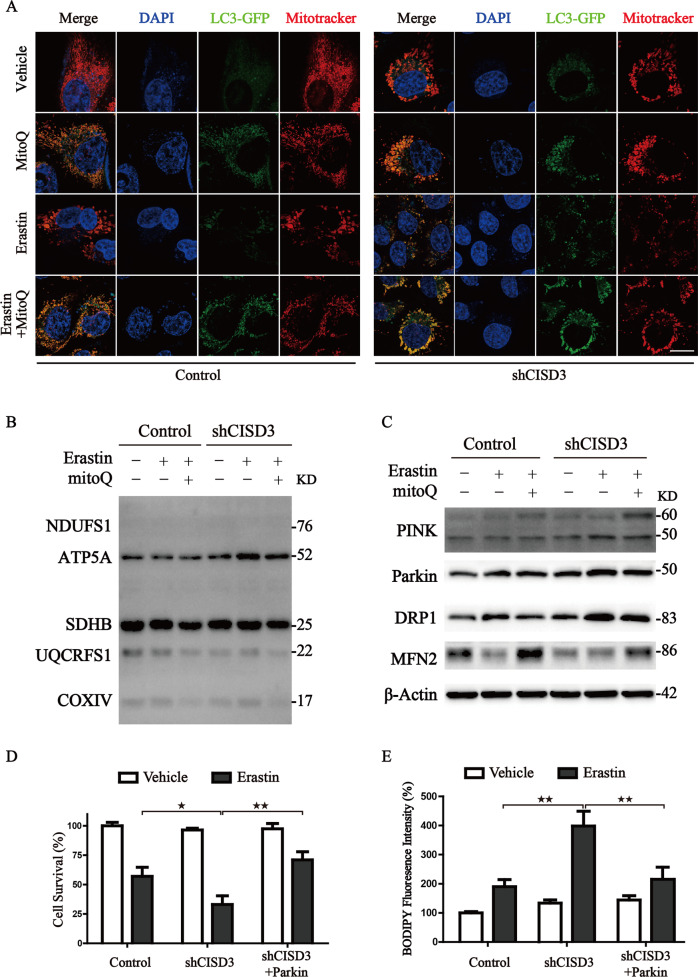
MitoQ ameliorates CISD3 associated ferroptosis via regulation mitophagy and the process of mitochondrial dynamics. **A** Cells were transfected with LC3-GFP, after indicated treatment (7.5 µM erastin; 0.5 µM mitoQ) for 4 h, cells were co-stained with Mitotracker. The images were taken by a laser confocal microscope. Scale bar: 10 μm. **B, C** Protein levels were analyzed by western blot in control and shCISD3 cells with the designed treatment. β-actin served as the loading control. **D** Cell viability was assayed using CCK8 kit in the indicated cells with or without 7.5 µM erastin treatment for 12 h. **E** Lipid peroxides were assayed by BODIPY staining in the indicated cells with or without 7.5 µM erastin treatment. *P < 0.05, **P < 0.01 versus control or between different groups.

Based on reports from previous studies, mitochondrial antioxidants mitoQ could protect mitochondrial injury through activating mitophagy [21, 22]. Therefore, we wondered whether mitoQ-mediated mitophagy could block ferroptosis by clearing the damaged mitochondria in the CISD3 knockdown cells. To test this hypothesis, the LC3-GFP transfected cells were subjected to the indicated treatments and co-stained with mitotracker. Through morphological analysis of mitochondria, CISD3 knockdown disrupted mitochondrial homeostasis and induced compensatory activation of mitophagy characterized by increased autophagosome–mitochondria fusion, which is in line with the aforementioned results in Fig. 4E. Remarkably, co-treatment with mitoQ further facilitated the removal of defective mitochondria through selective mitophagy (Fig. 6A). The results from western blot also showed that the expression of ETC proteins was upregulated in CISD3 knockdown cells under the treatment of erastin, while pretreatment with mitoQ could suppress the abnormally elevated ETC proteins by promoting mitophagy (Fig. 6B). Furthermore, CISD3 knockdown disrupted the process of mitochondrial dynamics. Pretreatment with mitoQ significantly blocked the mitochondrial fission and maintained the mitochondrial dynamics in the CISD3 knockdown cells. In addition, we found PINK1-Parkin pathway was activated and participated in the protective mitophagy process (Fig. 6C). Ectopic expression of Parkin in shCISD3 cells significantly blocked the ferroptotic cell death and lipid ROS generation under treatment of erastin (Fig. 6D, E). Thus, these results demonstrate that genetic and pharmacological activation of mitophagy can block ferroptosis by clearing the damaged mitochondria in shCISD3 cells.

### Free iron accumulation is associated with ferroptosis derived from CISD3 knockdown

**Fig. 7:**
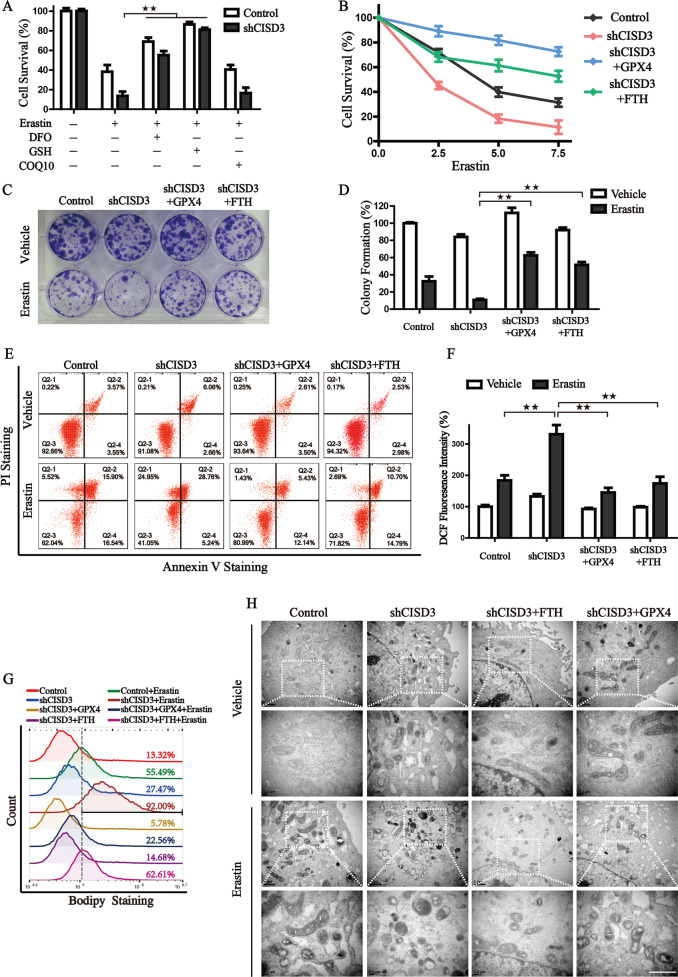
Free iron accumulation is associated with ferroptosis derived from CISD3 knockdown. **A** Cell viability assay was monitored in shCISD3 and control cells by CCK8 kit in the presence or absence of indicated molecules (7.5 µM erastin, 100 µM DFO, 0.5 mM GSH, and 10 µM COQ10). **B** Cell viability of CISD3 silenced cells with or without GPX4 or FTH expression were measured at different concentrations of erastin treatment for 12 h. Long-term cell viability was detected by colony formation assay under the exposure of 0.5 µM erastin for 14 days (**C**), and corresponding histograms are shown on the right (**D**). Cell death (**E**), cellular ROS (**F**), and cellular lipid peroxides (**G**) were detected by flow cytometry in indicated cells with or without erastin treatment through Annexin V-PI, DCF-DA, and BODIPY C11 staining, respectively. **H** The morphological changes of mitochondria were detected by transmission electron microscopy in the absence or presence of 7.5 µM erastin. Lower scale bars: 1 μm. *P < 0.05, **P < 0.01 versus control or between different groups.

To further investigate the mechanism of CISD3 knockdown-mediated ferroptosis, we tested the rescued effect of DFO, GSH, and COQ10, which represent the three main passways of ferroptosis, iron metabolism, GSH-GPX4, and FSP1-COQ10, respectively. Results revealed that administration of DFO and GSH, but not COQ10, could partially block erastin-induced ferroptosis in shCISD3 cells, suggesting that iron metabolism, GSH-GPX4 may play a role in ferroptosis mediated by CISD3 knockdown (Fig. 7A). We then transfected CISD3 silenced cells with GPX4 or FTH expression vector and found overexpression of GPX4 fully rescued CISD3 inhibition induced lipid ROS accumulation and ferroptosis under erastin treatment (Fig. 7B–G), but failed to reverse the morphological changes of mitochondria (Fig. 7H). This indicates that GSH−GPX4 functions as a downstream regulator of CISD3 modulated ferroptosis. The recovery phenotypes were also verified in FTH overexpression cells, and the results manifested that iron homeostasis regulation could modulate injuries derived from CISD3 knockdown, ameliorate the capacity of colony formation under erastin exposure, rescue the erastin induced accumulation of lipid peroxidation in shCISD3 cells, as well as prevent mitochondrial dysfunction (Fig. 7B–H). In summary, we propose that ferroptosis initiated via CISD3 knockdown is partly regulated by iron metabolism, and functions upstream of GPX4.

### CISD3 knockdown inhibits the growth of xenograft in vivo

**Fig. 8:**
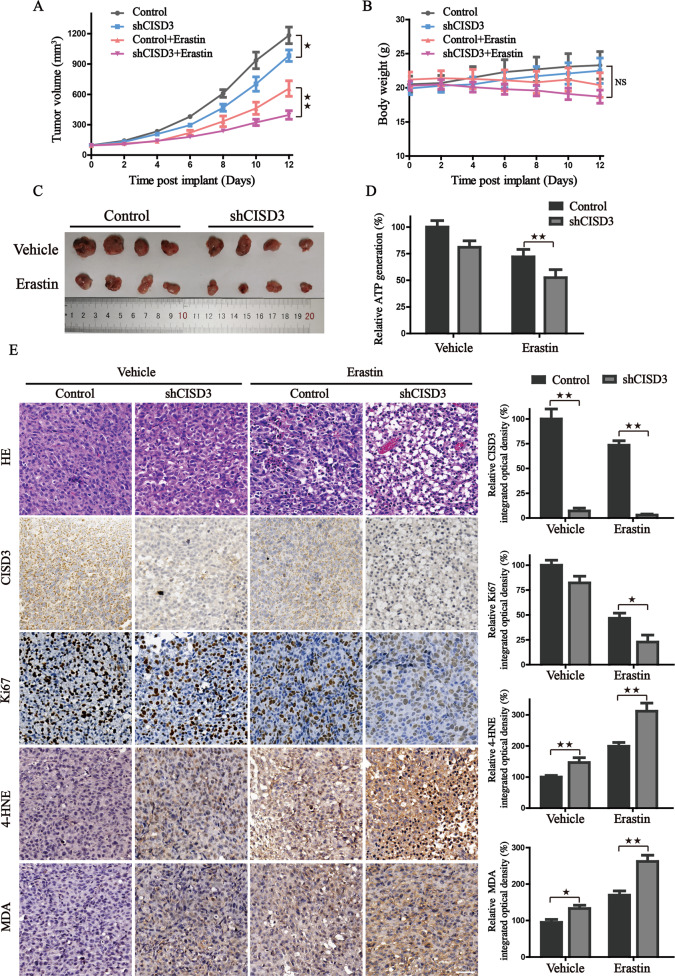
CISD3 knockdown inhibits in vivo growth of xenograft. **A** The growth curves representing the average size of xenografts in mice harboring HT1080 cells with altered CISD3 expression in the presence or absence of erastin administration. Tumor volume was monitored every two days and calculated using the formula: 0.5 × *a* × *b*^2^. **B** The body weight curves of mice in indicated groups. NS indicates no statistical significance. **C** The morphological images of dissected xenografts in indicated groups. **D** Xenografts in each group were lysed and the ATP content was analyzed. **E** H&E staining and IHC analysis for CISD3, Ki-67, 4HNE, and MDA in indicated tumor tissues. Representative images were showed and the statistical columns were quantified via relative integrated optical density (IOD) value and showed on the right. *P < 0.05, **P < 0.01 between indicated groups.

To evaluate the in vivo effect of CISD3 expression on tumor growth, the control, and CISD3 knockdown HT-1080 cells were subcutaneously implanted into the flank of immunodeficient nude mice. Compared to control shRNA cells, erastin effectively reduced the size of tumors formed by shCISD3 cells (Fig. 8A–C). Further, the results from H&E staining showed that the pathological structure of the xenografts was dramatically destroyed in CISD3 silenced tumor tissues under the treatment of erastin (Fig. 8E). CISD3 depletion locally diminished Ki67 expression, augmented MDA and 4-HNE accumulation, and decreased ATP production in xenograft tissues (Fig. 8D–E). These results are in line with the in vitro points that CISD3 inhibition impairs mitochondrial function, exacerbates lipid peroxidation, and represses the growth of cancer cells. Thus, CISD3 could serve as a promising therapeutic target, predisposing cancer cells to an increased risk of ferroptotic cell death.

## Discussion

Although the essential features of ferroptosis have been well-characterized, the detailed interaction and regulatory mode remain elusive. Therefore, more related proteins or regulators for ferroptosis should urgently be explored. In recent years, several iron−sulfur proteins have been identified associated with ferroptosis [23, 24]. Other studies also found that MitoNEET and NAF-1 play a vital role in the pathology of obesity, diabetes, heart disease, neurodegeneration, and cancer progression [17, 25, 26]. Limited information has been published on the homologous protein CISD3, which harbors one [2Fe−2S] cluster within a single polypeptide chain. This structural feature contributes to the high flexibility of the Fe−S cluster, making it readily available for redox reaction or electron transfer. Following previous studies, the homologous protein of CISD3 in *Caenorhabditis Elegans* exertes a regulatory function in physiological germline apoptosis [27]. A detailed investigation revealed that CISD3 knockdown elevates the accumulation of mitochondrial labile iron, as well as mitochondrial reactive oxygen production [17]. In this work, through pan-cancer analysis from TCGA, we revealed that expression of CISD3 is generally high in various human cancers. Patients with high CISD3 expression were associated with higher HR and poorer overall survival. Consistent with the bioinformatic analysis, we also verified that CISD3 participates in the proliferation of HT-1080 cells in vivo and vitro. Collectively, these findings demonstrate that CISD3 plays a role in the molecular biological characteristics of cancer.

The highly reductive and soluble Fe^2+^ serves as catalysts and cofactors, which participate in various biological processes and constitute the labile free-iron pool. Alteration of free iron level and iron metabolism proteins contribute to the regulation of ferroptosis sensitivity. A recent study also demonstrated that high dietary iron triggers the accumulation of lipid ROS and oxidative mitochondrial dysfunction, ultimately leading to ferroptotic cell death [28]. It is worth noting that, as highly soluble Fe^2+^ is indispensable for ferroptosis, it is evident that cancer cells are vulnerable to ferroptotic cell death for the increased iron demand compared with non-cancer cells. Therefore, targeting cellular catalyzed iron metabolism is a promising therapeutic strategy for the induction of tumorous ferroptosis. In our present study, we present evidence to strengthen that CISD3 is indeed a crucial regulator in cystine-deprivation ferroptosis. When CISD3 is depleted, free iron accumulates and lethal lipid peroxides overloads in the cells, thus become more sensitive to ferroptosis upon pharmacological inhibition of system Xc^–^. Moreover, the administration of iron chelator could partially rescue the ferroptotic cell death, and ectopic expression of FTH attenuated the mitochondrial dysfunction and lipid ROS accumulation in the shCISD3 cells under erastin treatment. Thus, we suggested that the dysfunction of iron metabolism partly contributes to the CISD3 mediated ferroptosis.

Mitochondria is essential for the fate of cells [7]. Mitochondrial morphology is a valuable parameter in predicting ferroptotic cell death, characterized as fragmentation, membrane density, cristae disappearance, and corresponding volume reduction, which is distinct from other forms of cell death. Once mitochondrial dysfunction occurs, large quantities of ROS, including hydrogen peroxide, superoxide anion, hydroxyl radical, and peroxynitrite are produced via mitochondrial metabolism [8]. Previously, a study found that cystine deprivation was associated with glutaminolysis fueling, enhanced TCA activity, and hyperpolarization of MMP, which resulted in reduced GSH levels and disordered oxidation [10]. Consistently, we found that CISD3 depletion presents a metabolic reprogramming toward glutaminolysis, which is required for the generation of sufficient lipid ROS to initiate ferroptosis. Both the inhibitors of glutaminolysis and the ETC process were capable of blocking the lipid peroxidation and ferroptotic cell death in shCISD3 cells when challenged by cystine-deprivation. Additionally, administration of mitochondria-targeted antioxidants SKQ1 and mitoQ exhibited more significantly protective effects. Therefore, it is conceivable that CISD3 protein is a key regulator of mitochondrial metabolic activity, and its depletion accelerates lipid peroxidation and ferroptosis by inducing mitochondrial dysfunction and facilitating ferroptotic cell death.

Mitophagy is an important cellular pathway to eliminate the damaged mitochondria for mitochondrial quality control and nutrients recycling. Its dysregulation is causally related to the production of ROS and programmed cell death [29, 30]. Minghui Gao and co-workers found that mitochondria-depleted cells were less sensitive to ferroptosis triggered by cystine-starvation or Xc^–^ inhibition [9]. However, the interplay between mitophagy and ferroptosis is still unclear. In our work, we found that CISD3 knockdown induced rupture of mitochondria and moderately activated mitophagy under the treatment of erastin. Interestingly, pretreatment with mitoQ, a mitochondria-targeted antioxidant, significantly increased the activity of mitophagy and blocked CISD3-mediated ferroptosis. Furthermore, enforced expression of Parkin in CISD3 knockdown cells prevented the ferroptotic cell death. Consequently, stimulation of PINK1-Parkin dependent mitophagy plays a protective role in the CISD3 mediated ferroptosis and could be a pathway of interest when considering methods to regulate the efficacy of ferroptosis.

Cancer cells are usually retained at an upper threshold of ROS than healthy cells [31]. The vulnerability and sensitivity of cancer cells to oxidative stress could be used as the “Achilles heel”, and the modulation of ROS level to induce ferroptosis in cancer cells has recently been popularized as a strategy for anticancer therapy [32]. Our current results illustrated a novel “Double-Hit Model” of shCISD3-initiated ferroptosis. One is that shCISD3 enhances the accumulation of free iron via iron starvation stress and results in lipid peroxidation; the other is that CISD3 depletion presents a metabolic reprogramming toward glutaminolysis to fuel the mitochondrial ETC, which results in oxidative stress and ferroptosis. Of note, this study firstly uncovered the role of CISD3 in molecular biological characteristics of cancer, and provided a potential target predisposing cancer cells to an increased risk of ferroptotic cell death.

## Supplementary information

Graphical abstract

Supplementary figure legends

Figure S1

Figure S2

Figure S3

## Data Availability

All data generated during this study are included either in this article or in the supplementary information files.
