# Supplementary figures and images for: CISD3 inhibition drives cystine-deprivation induced ferroptosis
Source: Cell Death Dis. 2021 Sep 8;12(9):839. doi: 10.1038/s41419-021-04128-2 (PMC8426496; doi:10.1038/s41419-021-04128-2)

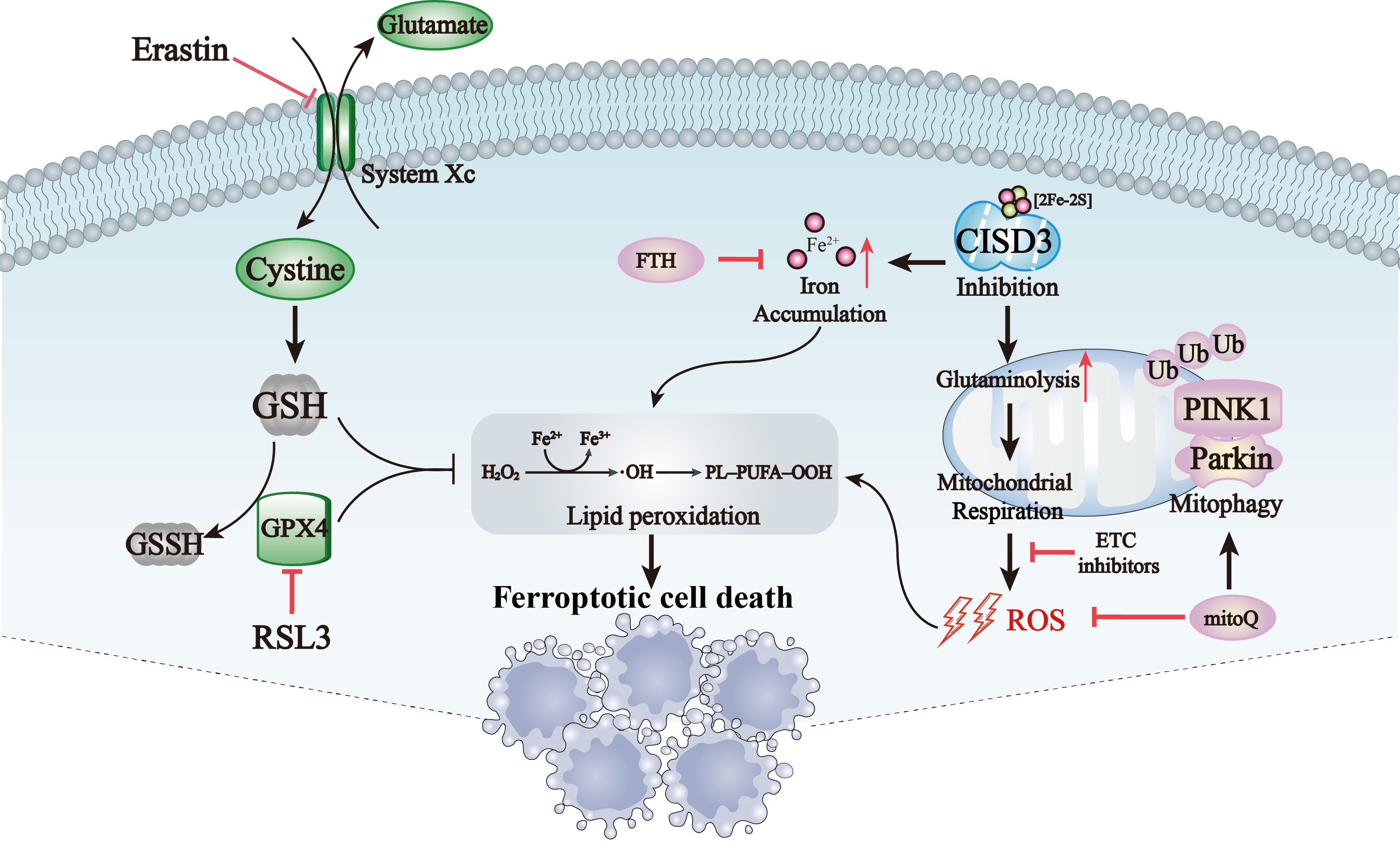

Supplement: Supplementary file 1 — Graphical abstract [file 41419_2021_4128_MOESM1_ESM.png]

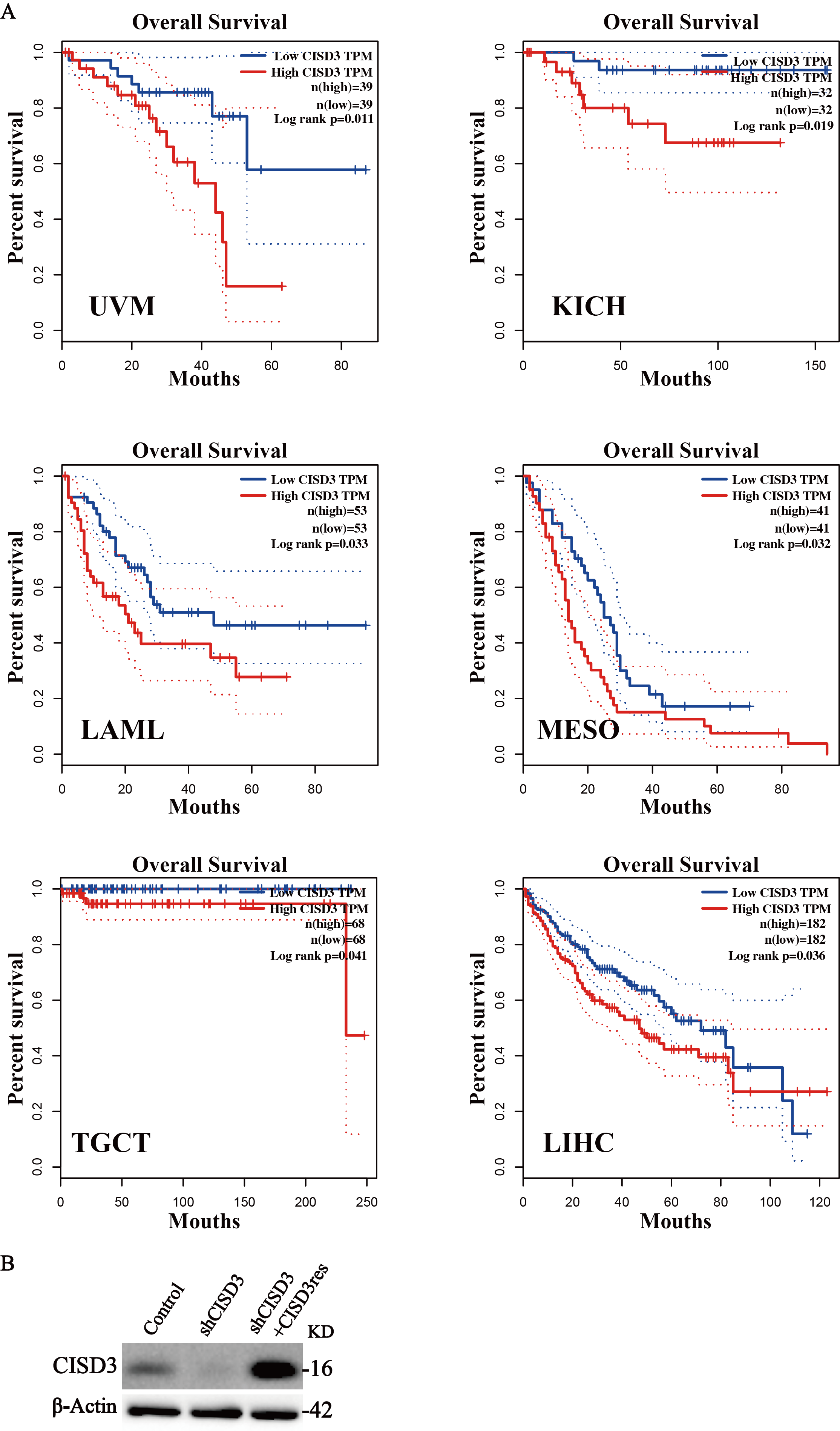

Supplement: Supplementary file 3 — Figure S1 [file 41419_2021_4128_MOESM3_ESM.png]

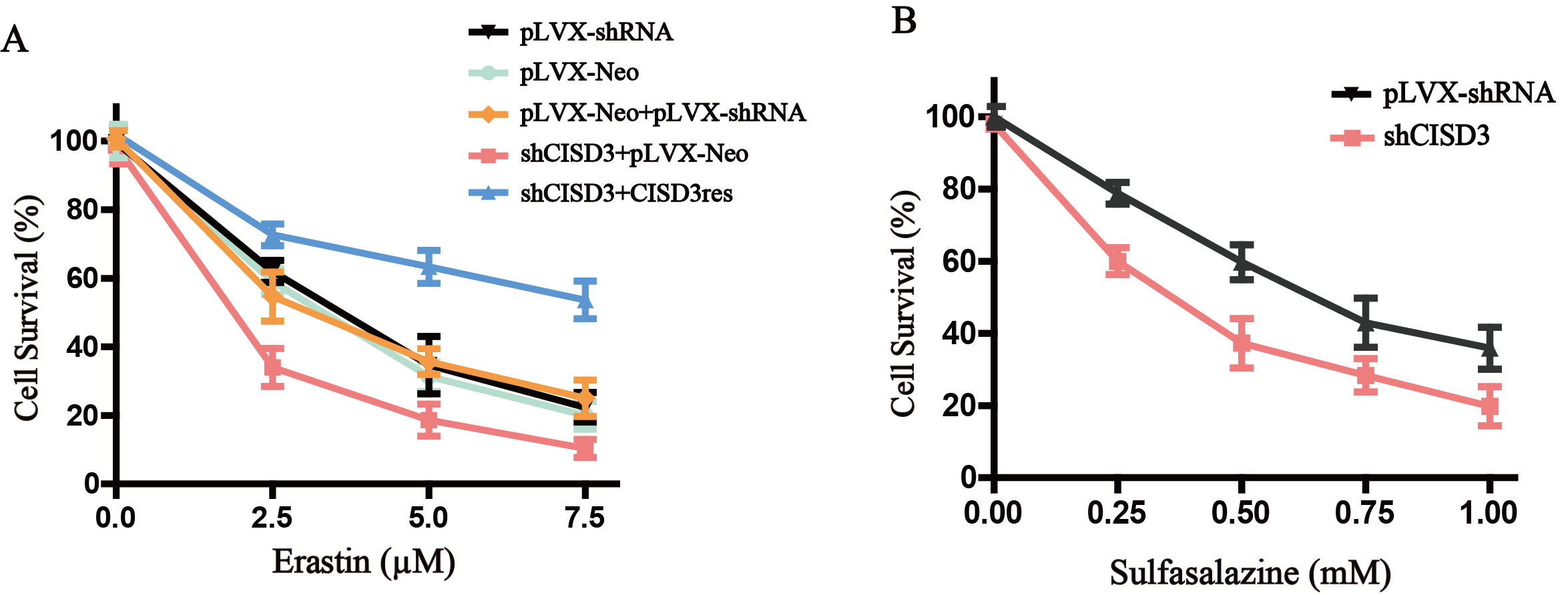

Supplement: Supplementary file 4 — Figure S2 [file 41419_2021_4128_MOESM4_ESM.png]

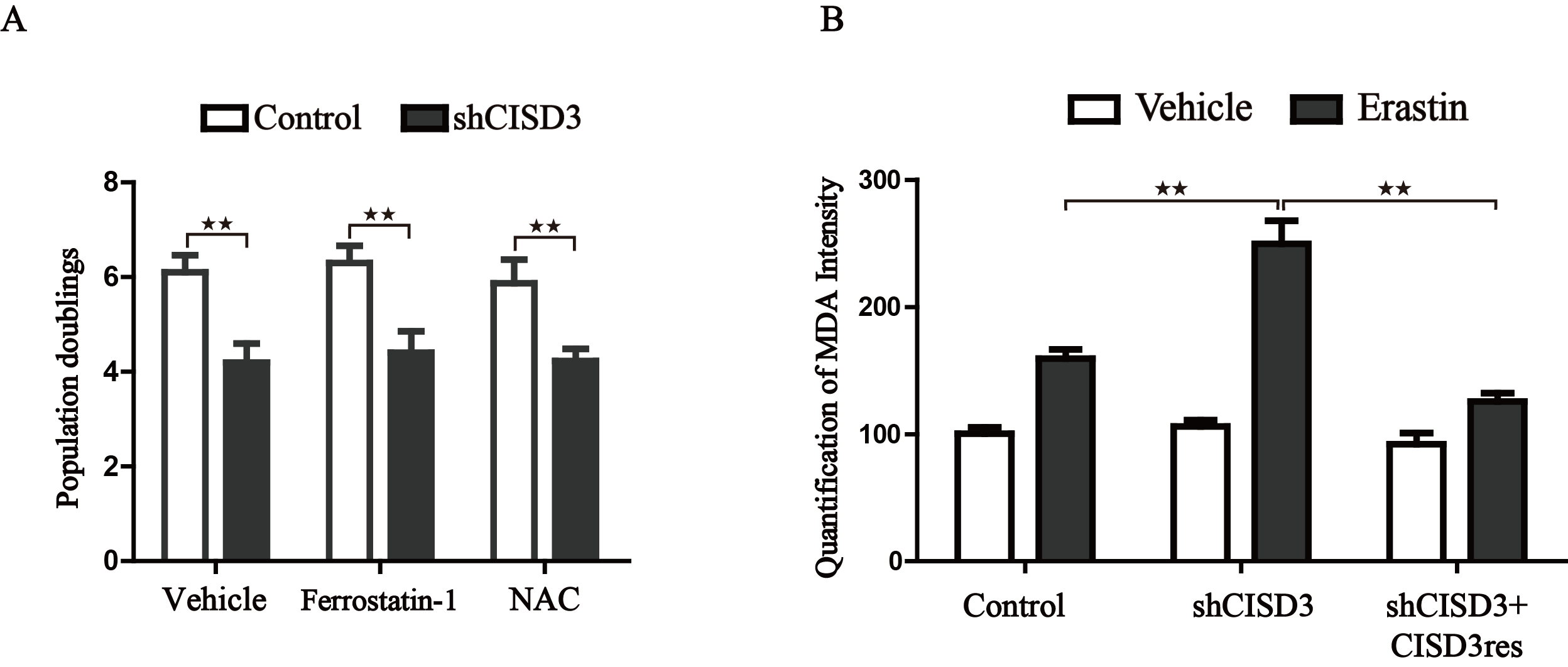

Supplement: Supplementary file 5 — Figure S3 [file 41419_2021_4128_MOESM5_ESM.png]
