# Supplementary material for: CISD3 inhibition drives cystine-deprivation induced ferroptosis
Source: Cell Death Dis. 2021 Sep 8;12(9):839. doi: 10.1038/s41419-021-04128-2 (PMC8426496; doi:10.1038/s41419-021-04128-2)
Supplement: Supplementary file 2 — Supplementary figure legends [file 41419_2021_4128_MOESM2_ESM.docx]

Fig. S1. CISD3 expression is associated with the progression of cancers

(A) High CISD3 expression level was correlated with poorer prognosis of OS in multiple tumor types from the TCGA datasets. A Log-rank test was used to compare the survival rates between groups. (B) The expression of CISD3 in the HT-1080 cells was detected by western blot assay. ^★★^*P < 0.01* between indicated groups.

Fig. S2. CISD3 facilitates cystine-deprivation induced ferroptosis

(A) The HT1080 cells with different empty vectors and CISD3res overexpression were subjected to the titration of erastin. Cell survival was measured by CCK8 assay. (B) Control and CISD3 knockdown cells were exposed to sulfasalazine. Cell survival was measured by CCK8 assay. ^★^*P < 0.05,* ^★★^*P < 0.01* versus control or between indicated groups.

Fig.S3 (A) The four-day proliferation assay of HT-1080 cells transfected with pLVX-shRNA or shCISD3 cultured with or without ferrostatin-1 and NAC. (B) The statistical histogram of MDA fluorescence in Fig.3G. ^★^*P < 0.05,* ^★★^*P < 0.01* versus control or between different groups.
